# Use of Antibiotics within the IMCI Guidelines in Outpatient Settings in Papua New Guinean Children: An Observational and Effectiveness Study

**DOI:** 10.1371/journal.pone.0090990

**Published:** 2014-03-13

**Authors:** Nicolas Senn, Patricia Rarau, Mary Salib, Doris Manong, Peter Siba, Stephen Rogerson, Ivo Mueller, Blaise Genton

**Affiliations:** 1 Vector Born Unit, PNG Institute of Medical Research, Madang (MAD), Papua New Guinea; 2 Health Intervention Unit, Swiss Tropical and Public Health Institute, Basel (BS), Switzerland; 3 University of Basel, Basel (BS), Switzerland; 4 Department of Medicine, University of Melbourne, Melbourne, Australia; 5 Dept. of Infections & Immunity, Walter & Eliza Hall Institute of Medical Research, Melbourne, Australia; 6 Centre de Recerca en Salut Internacional de Barcelona (CRESIB), Barcelona, Spain; National Institute of Medical Research, United Kingdom

## Abstract

**Introduction:**

There is a need to investigate the effectiveness and appropriateness of antibiotics prescription within the Integrated Management of Childhood Illness (IMCI) strategy in the context of routine outpatient clinics.

**Methods:**

Making use of a passive case detection system established for a malaria prevention trial in outpatient clinics in Papua New Guinea, the appropriateness and effectiveness of the use of antibiotics within the IMCI was assessed in 1605 young children. Main outcomes were prescription of antibiotics and re-attendances within 14 days for mild pneumonia, mild diarrhoea and uncomplicated malaria whether they were managed with or without antibiotics (proxy of effectiveness). Appropriateness was assessed for both mild and severe cases, while effectiveness was assessed only for mild diseases.

**Results:**

A total of 6975 illness episodes out of 8944 fulfilled inclusion criteria (no previous attendance <14 days+full medical records). Clinical incidence rates (episodes/child/year; 95% CI) were 0.85 (0.81–0.90) for pneumonia, 0.62 (0.58–0.66) for malaria and 0.72 (0.65–0.93) for diarrhoea. Fifty three percent of 6975 sick children were treated with antibiotics, 11% were not treated with antibiotics when they should have been and in 29% antibiotics were prescribed when they should not have been. Re-attendance rates within 14 days following clinical diagnosis of mild pneumonia were 9% (126/1401) when managed with antibiotics compared to 8% (56/701) when managed without (adjusted Hazard Ratio (aHR) = 1.00 (0.57–1.76), p = 0.98). Rates for mild diarrhoea were 8% (73/874) and 9% (79/866) respectively (aHR = 0.8 (0.42–1.57), p = 0.53).

**Conclusion:**

Non-adherence to IMCI recommendations for prescription of antibiotics is common in routine settings in Papua New Guinea. Although recommended, the use of antibiotics in young children with mild pneumonia as defined by IMCI criteria did not impact on their outcome. Better tools and new strategies for the identification of bacterial infections that require antibiotics are urgently needed.

## Introduction

Worldwide, the leading causes of death in children under five years (excluding perinatal mortality) are pneumonia, diarrhoea and malaria. These three diseases are responsible for an estimated 5 million deaths yearly, over 90% of which occur in Africa and other developing countries with limited resources. [1] [2] At a community level and in outpatient settings, the few available studies show that pneumonia, diarrhoea and malaria are similarly responsible for a high burden of morbidity in developing countries [3], [4], [5], [6], [7].

For decades, a high importance was given to malaria as a cause of fever, which has led to an overestimation of its burden and at the same time an underestimation of other diseases. [8], [9] Nowadays, case management strategy of sick children under five years in developing countries is usually based on clinical presentation of diseases (syndromes) rather than on aetiologies, as recommended by the World Health Organization (WHO), which developed the Integrated Management of Childhood Illness (IMCI) guidelines for this purpose. [10] The syndromic approach has some limitations such as the potential overlap of signs and symptoms between different diseases. [11], [12], [13] It remains unclear whether this overlap of signs/symptoms is because children are commonly infected with multiple pathogens, or because distinct diseases present with the same clinical picture [11], [12].

Although the IMCI strategy has been used for more than 20 years, few studies have investigated its effectiveness and appropriateness. Specifically, there is limited information on how well health workers comply with IMCI guidelines in routine practice, and on the impact of IMCI recommendations on health outcomes. [14], [15] Although most experts agree that the introduction of IMCI has improved the quality of care at limited cost, [16] IMCI has however some limitations such as the absence of objective diagnostic tools or no consideration of the local epidemiology. All may result in low specificity of IMCI algorithms, especially to identify bacterial infections that require antibiotics. [3], [15], [16], [17], [18], [19], [20] Therefore, many children might receive unnecessary antibiotics. Furthermore, it still remains debated how many children who receive antibiotics prescribed according to IMCI recommendations benefit from them. For example, a study from Pakistan demonstrated that children with mild pneumonia did not benefit from antibiotics. [21] This study was conducted in controlled conditions with defined inclusion and exclusion criteria. No study investigated the accuracy of diagnosis and the impact of appropriate treatment on health outcomes in routine practice.

Papua New Guinea (PNG) has a high burden of malaria in coastal areas, while pneumonia is the leading cause of admission in children under five years nationally. [22], [23], [24] Case management of sick children is almost exclusively syndrome-based using IMCI guidelines. In outpatient settings, few diagnostic tools are available and until 2011 microscopy or RDTs were rarely available in health facilities to confirm malaria infections. Thus, presumptive treatments with antimalarials and/or antibiotics are prescribed to cover common diseases such as pneumonia, malaria or otitis media. In PNG, as in many places, no data are available on the performance of the IMCI strategy in outpatient settings.

As part of a trial on malaria prevention, a passive case detection system was set up to record illness episodes in all study participants aged 3 to 27 months. All illness episodes were managed in accordance with the PNG IMCI guidelines, except for malaria, where treatment was guided by RDT instead of using presumptive diagnosis. [25] This study provides therefore a unique opportunity to investigate the appropriateness and effectiveness of antibiotics prescribed within IMCI.

## Methods

This work was carried out alongside a 3-arm trial of Intermittent Preventive Treatment for malaria in infants (IPTi) that included 1605 children 3–27 months old from June 2006 to June 2010 (www.ClinicalTrials.gov: NCT00285662) [26].

### Study Sites and Population

The study took place in two regions of PNG: Mugil (Madang Province) with 1125 study participants and in the Wosera (East Sepik Province) with 480 study participants. Each study site included approximately 20 villages, serviced by four major health centres: Mugil and Alexishafen in Madang area, and Kunjingini and Kaugia in Wosera. Several aid posts were also used for the morbidity surveillance. All IPTi preventive treatments were given at time of routine vaccination at 3, 6 and 9 months or of Vitamin A supplementation at 12 months following the PNG national expanded programme of immunization.

The study was carried out in accordance with Good Clinical Practice (GCP) and monitored by an independent external monitor. The study was approved by the PNG Medical Research Advisory Committee (MRAC number 05.20). Parents or carers were asked to fill a written consent form prior to enrolment of their child in the trial.

Children enrolled in the trial were randomly allocated to receive placebo or two different antimalarial drug regimes [sulphadoxine/pyrimethamine (single dose) with either 3 days of amodiaquine (SP-AQ3) or 3 days of artesunate (SP-AS3)]. The primary objective of the trial was the determination of the protective effect of the two IPTi regimens on malaria episodes.

### Morbidity Surveillance

Throughout the trial, a passive case detection system was maintained at the four study health centres’ outpatient clinics as well as at four aid posts to assure a continuous morbidity surveillance of study participants. This means that parents of study participants were asked to attend the study clinics free of charge for clinical management whenever their child was sick. They were seen by study nurses, often in collaboration with the health centre’s staff. No active follow-up of study participants was performed for acute diseases (such as regular home visits). In that perspective, this approach is more likely to reflect routine practice in PNG and thus avoid overestimation of the burden of diseases. The passive surveillance records collected were used to perform the present analysis.

Each illness episode was assessed by study staff using a standard case report form. Main signs and symptoms were systematically recorded using tick boxes. In case of history of fever in the past 48 hours or an axillary temperature >37.5°C, a RDT for malaria (ICT Combo®, South Africa) was performed and haemoglobin (Hb) level measured using a portable Hemocue 201® machine (Angelholm, Sweden). Malaria treatment decision was based on the RDT result, and only children with a positive result were treated with artemether/lumefantrine, irrespective of the species. The children with moderate to severe anaemia (Hb<8 g/dl) received iron supplementation at the dose of 5 mg/Kg of ferrous sulphate for 6 weeks. Pulse oximetry was implemented in December 2007 in Mugil study clinic (Madang site) and used systematically to assess sick study participants from that date onwards. It was recommended to clinical staff that they should consider admitting the child if oxygen saturation dropped below 94%. All other illnesses were treated according to the standard treatment guidelines of PNG, which is based on IMCI. [27] Children were also assessed for danger signs or symptoms and referred to the health centre for admission when necessary. If children attended the health centre when study staff were not on duty, they received care from the regular health centre nursing staff. As they did not have the capacity to perform RDTs or other blood tests, treatment was delivered on presumptive diagnosis only. On site, study nurses recorded their presumptive diagnosis and provided treatment. All records were cross-checked by the study clinicians who confirmed the likely diagnosis. Both study nurses and clinicians could record as many as 3 different diagnoses for each episode.

A severe disease was defined as an illness episode with potential life-threatening features such as respiratory distress (nasal flaring or chest indrawing), impaired consciousness or severe pallor. At least one danger sign or symptom needed to be present for an episode to be classified as severe disease. A child with criteria for severe disease was not necessarily admitted for the following reasons: parents refused admission; health staff decided not to admit the child according to their personal judgment or because they did not identified properly danger signs and symptoms.

### Study Staff

The medical staff was composed of about 20 nurses and community health workers (CHW). They were specifically trained for malaria and anaemia management in line with the trial’s requirements (diagnosis and treatment). [26] For all other diseases (e.g. pneumonia, diarrhoea or otitis media), they were asked to follow the national recommendations. No specific training was provided for IMCI, except a broad refresher training of one day per year on the basic management of sick children, including measurement of respiratory rate, evaluation of danger signs and key features of IMCI. Therefore, the care provided for non-malarial illness episodes was very close to what is usually performed in government health facilities, except that there was no shortage of drugs and consumables.

### Laboratory Procedures

A finger prick blood sample was used to perform a rapid diagnostic test (RDT) for malaria on site (to guide malaria treatment) and to record haemoglobin concentration. A blood slide was prepared for expert microscopy that was performed elsewhere.

### Coding of Diagnosis and Treatments

Using records of signs and symptoms (tick boxes) of the passive case detection forms, objective diagnoses were generated by computer according to IMCI definitions of diseases (e.g. mild pneumonia = cough and/or difficulties in breathing+respiratory rate >50 if <1 year or respiratory rate >40 if >1 year without sign/symptom of severity). For the present study, three diseases were explored with different levels of severity according to IMCI: 1) mild and moderate-to-severe pneumonia, 2) mild and severe diarrhoea and 3) uncomplicated and severe malaria. **Table 1** displays the detailed definitions of the different syndromes based on IMCI criteria. We use the terminology of the PNG-specific IMCI guidelines that was in use at the time of the study. **Table 1** includes the correspondence with the WHO-IMCI classification.

**Table 1:** Definition of diseases (including the use of RDT for malaria).

	Criteria	Standard treatment	WHO IMCI terminology
**Respiratory infections**			
** Acute Respiratory Infection (ARI)**	Cough and/or difficulties in breathing		**ARI**
** Upper Respiratory Tract** **Infection (URTI)**	Cough and/or difficulties in breathing AND RR<50(<1 Y) RR<40 (>1 y) AND No respiratorydistress (chest indrawing, cyanosis, nasal flaring)	Paracetamol	**no pneumonia/cough and cold**
** All pneumonia**	Cough and/or difficulties in breathing AND RR>50(<1 Y) RR>40 (>1 y)		**n/a**
** mild pneumonia**	Cough and/or difficulties in breathing AND RR>50(<1 Y) RR>40 (>1 y) AND No respiratorydistress (chest indrawing, cyanosis, nasal flaring)	Amoxicillin 5 days	**non-severe pneumonia**
** Severe pneumonia**	Cough and/or difficulties in breathing AND RR>50(<1 Y) RR>40 (>1 y) AND Respiratory distress(chest indrawing, cyanosis, nasal flaring)	Benzylpenicillin im orchloramphenicolim 10 days, admit	**severe and very severe pneumonia**
**Diarrhoea**			
** All diarrhoea**	Diarrhoea and/or vomit		**n/a**
** Mild diarrhoea**	Diarrhoea and/or vomit AND No dehydration(slow skin pinch, sunken eyes)	more hydration+breastfeed	**No or some dehydration**
** Severe diarrhoea**	Diarrhoea and/or vomit AND dehydration(slow skin pinch, sunken eyes)	ORS or iv fluid+/− admit	**severe dehydration**
**Malaria**			
** All malaria**	Fever and/or history of fever in past 48 hAND positive RDT (any species)		**n/a**
** Uncomplicated malaria**	Fever and/or history of fever in past 48 hAND positive RDT (any species ANDNo danger sign	Artemether/lumefantrinefor 3 days	**n/a**
** Severe malaria**	Fever and/or history of fever in past 48 hAND positive RDT (any species) AND At leastone danger sign	Artemether im+sulfadoxine/Pyrimethamine+admit	**n/a**

Ten distinct treatment categories were created: 1) artemether/lumefantrine, 2) iron+folic acid, 3) albendazole, 4) amoxicillin, 5) co-trimoxazole, 6) paracetamol, 7) tinidazole, 8) other antibiotics (erythromycin, benzylpenicillin im, chloramphenicol and ceftriaxone iv/im), 9) other antimalarial drugs (artemether, quinine, amodiaquine, sulphadoxine/pyrimethamine, chloroquine, 10) other drugs.

### Study Procedure

Illness episodes were included in the study if they fulfilled the following inclusion criteria:

- IPTi study participant
- No illness episode in the past 14 days (to avoid counting the same episode twice)
- Full clinical records (signs and symptoms available)

The appropriateness of the prescription of antibiotics by health staff was assessed for main diagnoses according to the IMCI recommendations, including both severe and mild disease episodes.

The effectiveness of antibiotics was investigated for mild diseases only, by calculating the rate of re-attendance within 14 days and the outcome upon re-attendance (death, severe illness or mild disease) for specific syndromes depending whether they were treated with antibiotics or not. Severe cases were excluded from this analysis for several reasons: i) severe cases (i.e. with danger signs) had to be admitted according to recommendations and managed systematically with intravenous antimalarial and antibiotics irrespective of the symptoms and signs; ii) the low prevalence of severe cases, which are almost all treated with antibiotics, prevent the investigation of the effectiveness because of the absence of a valid control group; iii) re-attendance rates was used as a proxy for effectiveness, but this is not a valid measure for assessing effectiveness in severe cases, as they should be all admitted in the first place and would therefore not re-attend; iv) there is no debate yet on the benefit of treating all severe cases with antibiotics.

This analysis was possible as syndromes were not necessarily correctly identified by health staff and therefore some children did not receive antibiotics when they should have.

### Statistical Analysis

All data were double entered using FoxPro software. Data analyses were performed using STATA software (version 12.0). More than one diagnosis was possible for each illness episode. Incidence rates were calculated using a negative binomial regression model. Cox regression model was used to calculate hazard ratios for the impact of antibiotics on pneumonia, diarrhoea and malaria (excluding severe diseases). The model was adjusted for potential confounders (markers of severity of respiratory infections): respiratory rate (continuous), temperature (continuous) and pulse oximetry (continuous). Kaplan-Meier survival curves were also calculated and displayed as time to first re-attendance over a 14-day observation period following an initial pneumonia, malaria or diarrhoea. All rates are displayed with a confidence interval of 95%. Differences between rates were assessed using a chi square test.

## Results

### Baseline Characteristics

Out of 1605 children 1512 (94%) presented at least 1 illness episode (Madang = 1053 and Maprik = 459). The morbidity surveillance recorded a total of 8943 illness episodes (Madang = 5977 and Maprik = 2966). Three hundred seventy one severe illnesses and 13 deaths occurred during the study. Overall, 2903 illness episodes occurred in the placebo group, compared to 3038 in the SP-AQ group and 3002 in the SP-AS arm.

Inclusion criteria were fulfilled for 6975 illness episodes (no visit in the previous 14 days and full medical records available). **Table 2** displays the danger signs and symptoms, clinical and laboratory features. As shown, 80% of the illness episodes presented with fever/history of fever and 76% had cough. No significant difference was observed between the different IPTi treatment groups (data not shown).

**Table 2:** Clinical and paraclinical features of 6975 illness episodes.

	%	(N)
**Danger signs**		
Unable to eat	0.6	44
Vomit everything	0.5	38
Unconscious/drowsy	1.4	95
Respiratory distress	1.7	117
Fits	0.8	58
Inability to sit up	0.2	12
Neck stifness	0.1	8
Severe Dehydration	0.7	49
Pale with heart rate >160 or oedema	0.2	17
**Main signs, symptoms**		
Fever (temp>37.5°/hist of fev)	80.3	5603
Palor	2.7	185
Cough	76.4	5332
Difficulties in breathing	14.9	1037
Running nose	46.2	3207
Diarrhoea	21.1	1474
Abdominal pain	2.7	191
Vomit	10.2	713
Ear pain	0.6	44
Skin rash	0.3	13
Skin abscess	1	67
Pus out of the ear	2.6	183
Respiratory rate >50	19.9	1390
**Laboratory findings**		
Pulse O2<94%	3.4	54*
Pulse O2<93%	2.7	42*
Mean Hb level (g/L)	9.6	–
Hb <8 g/L	11.1	626**

*only 1582 records with pulse oxymetry measured.

**only 5644 records with Hb measured.

### Description of Main Diseases According to IMCI Criteria and their Incidence Rates

Out of the 6975 illness episodes, 76.5% (5335) were ARI, including 44.7% (3116) URTI, 30.1% (2102) mild pneumonia and 1.7% (117) severe pneumonia. 25.6% (1788) had diarrhoea, including 24.9% (1740) mild diarrhoea and 0.7% (48) severe diarrhoea. 25.1% (1750) had malaria, including 23.6% (1643) uncomplicated malaria and 1.5% (107) severe malaria. 10.2% (710) could not be classified in one of these syndromes. In this category, there were mainly children with running nose as single symptom (which cannot be classified as URTI according to IMCI), skin infections and fever without any other accompanying symptom.

The incidence rates (IR, episode/child/year) were 0.85 (95% CI, 0.81–0.90) for mild pneumonia, 0.72 (95% CI, 0.65–0.93) for mild diarrhoea and 0.62 (95% CI, 0.58–0.66) for uncomplicated malaria. No differences were found in the incidence of non-malarial illnesses between the placebo and IPTi intervention arms. Indeed, incidence rates for all pneumonia in children 3–15 months old were very similar in all arms with 0.97 (95% CI, 0.89–1.06) in the placebo group, 1.00 (95% CI, 0.92–1.09) in the SP-AQ3 group and 0.98 (95% CI, 0.90–1.07) in the SP-AR3 group, p-values for incidence rate ratios (IRR) were 0.63 and 0.87 respectively. For diarrhoea (severe included), the incidence rates from 3–15 months were similar in all arms with: 0.94 (95% CI, 0.86–1.02) for placebo, 0.92 (95% CI, 0.84–1.0) for SP-AQ3 and 0.89 (95% CI, 0.81–0.99) for SP-AS3, p-values for IRR were 0.85 and 0.51 respectively. By contrast, the IPTi has proven to be effective on malaria as described elsewhere. Main results showed that the incidence of clinical malaria between the first dose at 3 months and 15 months was 0.85 per year-at-risk overall and 0.22 and 0.68 for P. falciparum and P. vivax, respectively. In the intention-to-treat analyses, the protective efficacy against all cases of malaria was 29% ([95% CI, 0.10, 0.43], p = <0.001) in the SP-AQ3 group and 12% ([95% CI, −0.11, 0.30], p = 0.12) in the SP-AS3 group [26].

### Use of Antibiotics and Appropriateness of Prescription

Out of the 6975 illness episodes, 53.6% (3741) were treated with antibiotics (at least one of amoxicillin, co-trimoxazole, erythromycin or i.m. penicillin or ceftriaxone) and 25.1% (1750) received antimalarial drugs (97.6% of whom had a positive RDT result). Among the children who were treated for mild pneumonia with antibiotics, 69% received amoxicillin only, 9% received co-trimoxazole only, 16% received other antibiotics and 6% received a combination of amoxicillin and other antibiotics.

**Figure 1:**
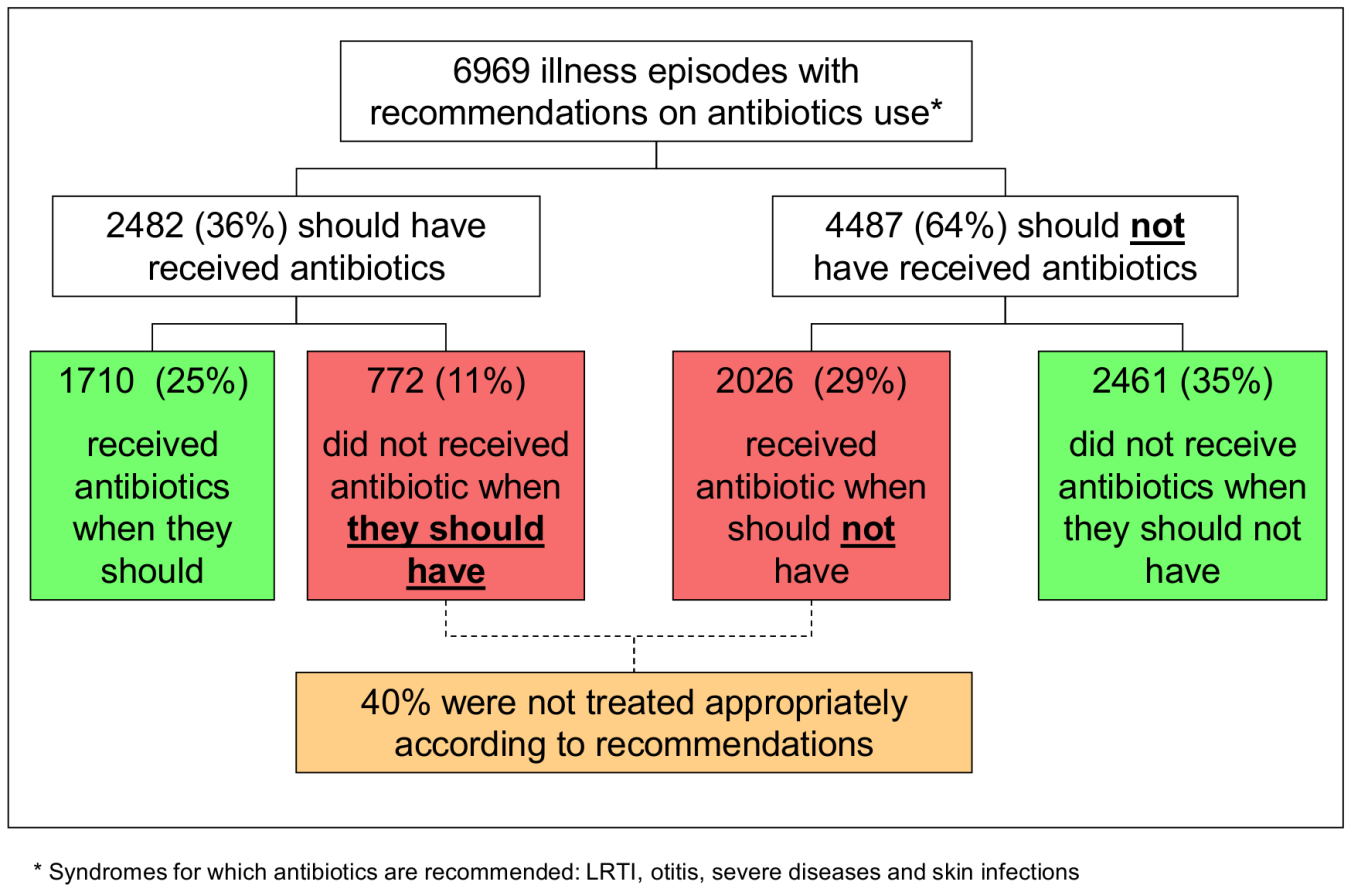
Appropriateness of antibiotics’ use according to the IMCI recommendations.

The overall appropriateness of antibiotics use was evaluated in 6969 illness episodes for which clear recommendations exist in the IMCI whether to prescribe or not antibiotics. According to these data, 40% of children were not treated appropriately, of whom 11% did not receive antibiotics when they should have and 29% received antibiotics when they should not have. (**Figure 1**).

Among the 2102 children with an objective diagnosis of mild pneumonia, 66.7% (1401) received (appropriately) antibiotics. Of 532 children who presented with criteria for both mild pneumonia and uncomplicated malaria (based on positive RDT), only 40% (213) were prescribed antibiotics. In contrast, when they had criteria for pneumonia only (without malaria), 75.7% (1188/1569) were prescribed antibiotics (p<0.001). Among 117 children with a diagnosis of severe pneumonia, 96.6% (113) received antibiotics, of whom 75.2% (88) were treated with parenteral antibiotics as per WHO guidelines and 49.6% (58) were admitted.

### Effectiveness of Antibiotics for Mild Pneumonia, Uncomplicated Malaria and Mild Diarrhoea

**Figure 2:**
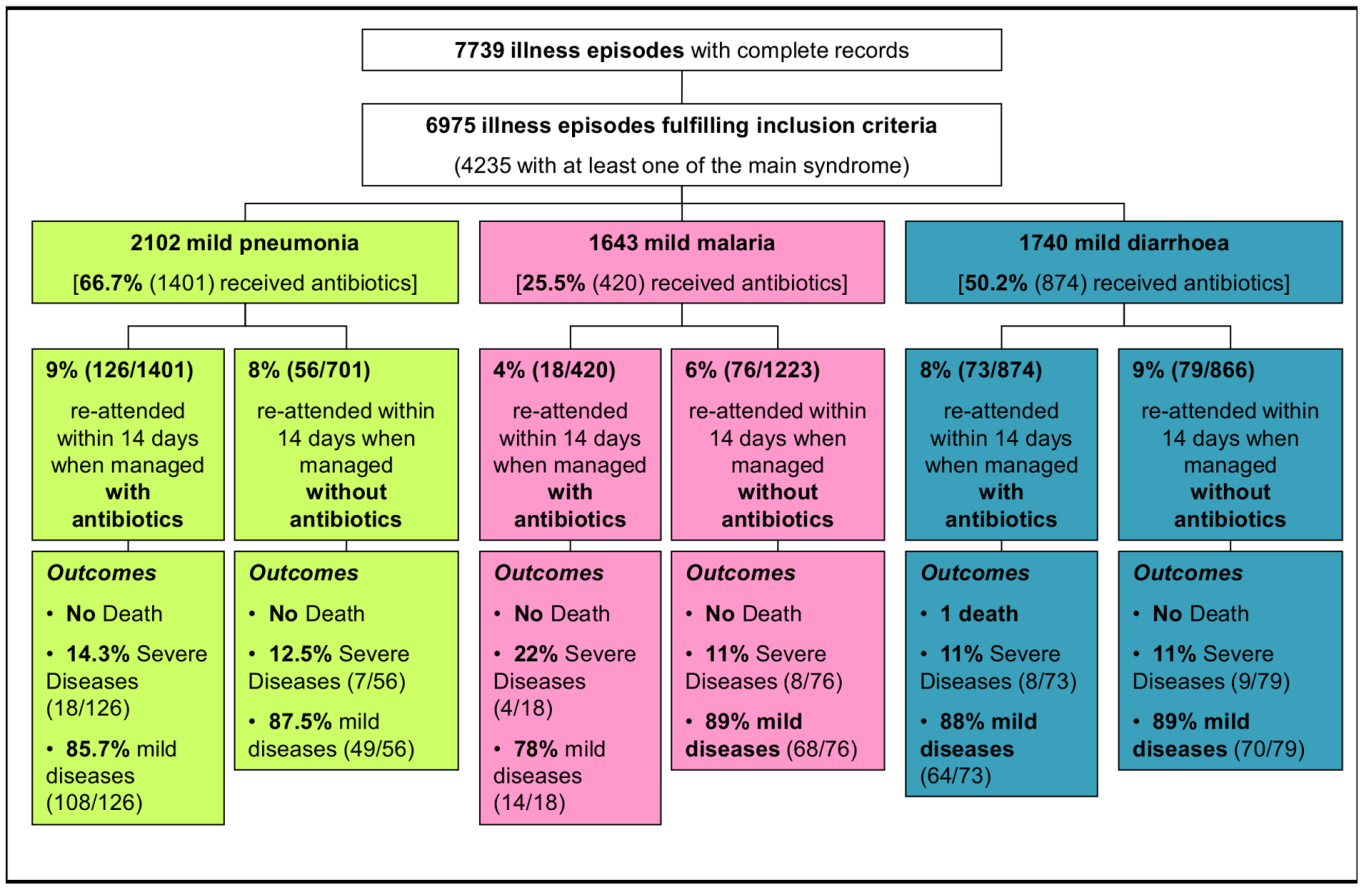
Re-attendance rates within 14 days and outcomes for the most common mild syndromes according to the prescription of antibiotics.

**Figure 3:**
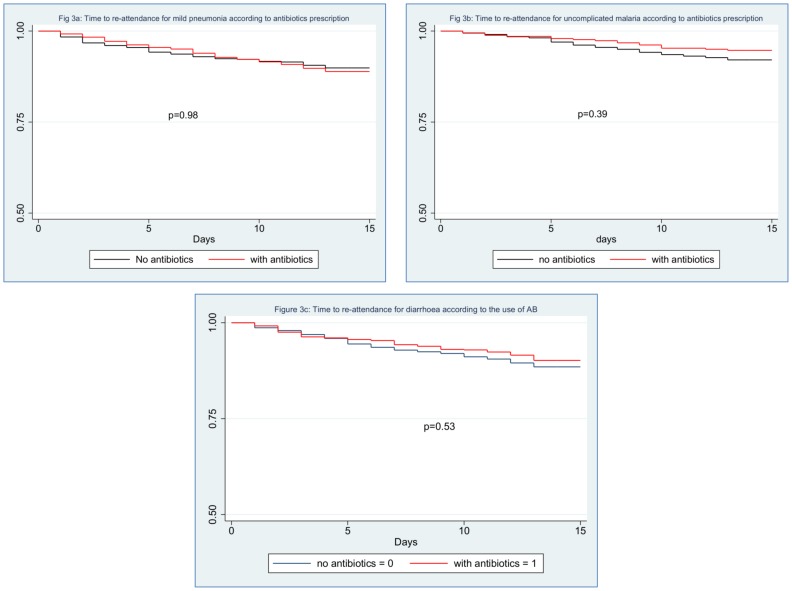
Kaplan–Meier estimates of the proportion of patients with mild pneumonia (3a), mild malaria (3b) or gastroenteritis (3c) re-attending the clinics within 14 days whether they received antibiotics (red line) or not (blue line).

Re-attendance rate within 14 days following an objective diagnosis of mild pneumonia (IMCI criteria) was 9% when treated with antibiotics compared to 8% when they did not receive any antibiotics, unadjusted Hazard Ratio (HR) was 1.09 (95% CI 0.80–1.49, p = 0.59). For uncomplicated malaria, re-attendance rates were 4% when they received antibiotics vs 6% when they did not [HR 0.66 (95% CI 0.39–1.10, p = 0.11)]. For diarrhoea, re-attendance rates were 8% when they received antibiotics and 9% when they did not [HR 0.85 (95% CI 0.61–1.16, p = 0.3)]. When adjusting for potential confounders including severity parameters (respiratory rate, pulse oximetry and fever), the adjusted HR (aHR) for mild pneumonia was 1.00 (95% CI, 0.57–1.76, p = 0.98), for mild malaria 0.70 (0.31–1.59, p = 0.39) and for mild diarrhoea 0.81(0.42–1.566, p = 0.53). **Figure 2** displays the re-attendance rates and outcomes within 14 days following the initial consultation for mild pneumonia, uncomplicated malaria and mild diarrhoea according to the prescription of antibiotics. **Figures 3a, 3b, 3c** displays the Kaplan-Meyer survival curves for these three diseases whether they were managed with or without antibiotics. There was no significant difference between the curves with or without antibiotics for all three diseases.

## Discussion

The aim of this prospective study was to explore the appropriateness and effectiveness of prescription of antibiotics in routine outpatient settings of Papua New Guinea in the frame of the IMCI recommendations.

### Overtreatment with Antibiotics

A high rate of antibiotic prescription was observed in the cohort, with 54% of cases treated (including mild and severe cases) when a maximum of 36% should have received antibiotics according to IMCI criteria, This is high compared to other settings, where it is usually reported that approximately 20% of patients require antibiotics when the diagnosis is made by highly skilled clinicians. [7], [15] This high rate of prescription may be due to difficulties in accurately recognizing or measuring signs and symptoms by health staff. For example, it is frequently observed that respiratory rate is not properly assessed and therefore tachypnoea often overestimated. In our setting, despite the official recommendation of counting over 1 minute, most respiratory rates were even numbers suggesting that health workers are not counting according to standard operating procedures. (Senn & Rarau, personal communication) This is especially worrying as tachypnoea is a cornerstone for the diagnosis of pneumonia and hence for the prescription of antibiotics. Improving skills and eventually finding new tools for the accurate determination of the respiratory rate would be an important step towards more rational use of antibiotics.

### Appropriateness of Antibiotics Prescription

A poor compliance to guidelines was observed as 40% of patients were not treated according to the recommendations (29% received antibiotics when they should not have and 11% did not receive antibiotics when they should have). Apart from the inaccurate clinical judgment of health staff, other factors might influence their decision. In our study, we observed that the prescription of antibiotics was strongly influenced by the result of the RDT for malaria in feverish patients. Indeed, we observed that in children presenting with criteria for mild pneumonia (who should receive antibiotics), only 40% were treated when the RDT was positive, compared to 76% when the test was negative. On one hand, it is reassuring to observe that clinicians are influenced in their clinical judgment and treatment attitude by an objective test. On the other hand, considering only one diagnosis may have potential serious consequences for children, as it might leave children with bacterial infections untreated. This may be the downside of the introduction of RDT in routine clinics. Indeed, improving the diagnosis of malaria may have adverse effects for the management of other diseases, which is in line with findings by Bisoffi et al. in Africa [28]. This will need to be addressed when redesigning guidelines.

It is finally reassuring to observe that almost all children (97%) with criteria for a severe pneumonia received antibiotics.

### Effectiveness of Antibiotics

One might worry that such a high rate of non-compliance to standard IMCI recommendations might leave a lot of children with potential bacterial pneumonia untreated and might re-attend with a more severe clinical presentation associated with poor outcomes. However, our data indicate that it is in fact not the case. Indeed, the rate of re-attendance following a visit for mild pneumonia, which can be considered as a proxy of the effectiveness of the antibiotics, was similar, irrespective of antibiotic prescription (9% when they received antibiotics and 8% if they did not, adjusted hazard ratio of 1.0). This is well in line with recent findings in Pakistan where the use of amoxicillin was not found to provide any benefit for non-severe pneumonia as defined by WHO. [21] More generally and from a policy perspective, IMCI guidelines are not only aiming at reducing under five mortality by providing efficient strategies to treat acute severe cases but also to prevent the worsening of mild diseases. In that perspective, it was postulated that the broad prescription of antibiotics should mitigate the risk of poor outcomes. Our study looked specifically at that aspect in an outpatient setting and provides solid evidence to demonstrate that treating broadly mild diseases with antibiotics does not help prevent severe diseases. Recently, WHO and UNICEF, aware of the lack of specificity of the current IMCI guidelines, revised their recommendations and proposed a new classification of respiratory infections, notably merging non-severe and severe pneumonia into “pneumonia” and “severe pneumonia” being reserved for children with danger signs. The results of the present study validate and support this revision.

The absence of difference in the re-attendance rates is probably due to the fact that the vast majority of mild pneumonia episodes have a viral aetiology rather than bacterial, highlighting the lack of specificity of the clinical criteria to identify bacterial pneumonia. This was confirmed by a microbiological investigation made on nasal swabs in a sub-sample of these children. The rapid antigen diagnostic tests for viral pathogens showed that more than half of children with mild clinical pneumonia were positive for at least one of the four most common respiratory viruses: Influenza A&B, *Adenovirus* and Respiratory Syncytial Virus (Rarau et al. personal communication). Similar findings have also been published in other countries. [7] The fact that mortality was very low and most pneumonias were treated with amoxicillin (instead of co-trimoxazole), speaks against the hypothesis that similar outcomes between antibiotics users and non-users were due to the high prevalence of resistant pathogens [29].

The diagnosis of pneumonia is more complex than that of malaria as no reliable and easy-to-use tests are available to identify respiratory infections that require antibiotics. It has been recognized that pneumonia is often associated with virus detection and that there is a complex and poorly understood interplay between viruses and bacteria in the development of pneumonia. [30] Considering the dramatically high burden of respiratory illnesses, especially in very young children, the development of more accurate diagnostic strategies able to identify children with pneumonia requiring antibiotics should be on the top list of priorities. [7] The potential benefits of the use of RDT to document viral pathogens such as RSV or *Influenza* need to be investigated. Beyond the aetiology of respiratory infections, it would be also valuable to develop tests able to identify children with potentially severe (invasive) diseases, which require specific supportive care. In that regard, inflammatory markers such as pro-calcitonin (PCT) can be valuable tools. Recent reviews by Schuetz et al. [31], [32] have shown that PCT-guided prescription of antibiotics for ARI was safe and reduce substantially the consumption of antibiotics. They also noted that evidence is lacking on usefulness of PCT in settings outside Europe. C - reactive protein (CRP) can be also another valuable biomarker but results of randomized trials and meta-analysis are more conflicting. [33], [34], [35], [36] Finally, more experimental tools such as analysis of exhaled gases could be also considered [37]. New recommendations that integrate clinical assessment and biomarkers for the identification of cases that require antibiotics need to be seriously considered. However, further pragmatic research is needed to explore the most efficient, safe and cost-effective approach in children in resource-limited countries.

Similarly to what was observed with mild pneumonia, antibiotics did not provide any benefit for diarrhoea or malaria in our cohort, which supports the current WHO recommendations of not treating mild gastroenteritis and uncomplicated malaria with antibiotics.

### Limitations of the Study

The main limitation of this study is that the clinical management of malaria episodes was performed in ideal conditions because it was conducted alongside a malaria prevention trial (i.e. treatment of only RDT positive cases). While RDT-based malaria treatment was not part of the PNG malaria treatment guidelines when the study was conducted, there was rapid roll-out and rapid up-take of RDTs [38] and the situation in our study therefore increasingly resembles the situation in almost all malaria endemic countries including PNG. On the other hand, for all other diseases, the recommendations made to the study nurses were identical to the national standard treatment protocols. Another limitation is the lack of randomization for the use of antibiotics, which might have led to selection biases. However, even when we adjusted for potential confounders of disease severity (such as respiratory rate or pulse oximetry), no significant difference was observed whether illness episodes were treated with antibiotics or not.

## Conclusions

This study shows that adherence to IMCI recommendations for prescription of antibiotics was poor in routine outpatient settings of Papua New Guinea and was strongly influenced by RDT results for malaria, independently of the clinical presentation. However, the management of mild pneumonia with antibiotics based on IMCI guidelines did not change health outcome in terms of re-attendance and complications when compared to no antibiotics. This absence of effectiveness of antibiotics is certainly due to the lack of specificity of the present recommendations to differentiate children with true bacterial diseases and those with viral infections. It is urgently needed to develop better strategies to improve the identification of pneumonia and other syndromes that require antibiotics or more intensive clinical care.

## Acknowledgments

We would like to warmly thank all study nurses of the IPTi trial who looked after the data collection as well as after the study participants. We would like also to thank all the laboratory staff of the PNG IMR who looked at some many slides and analysis.

Finally our deep gratitude goes to all the study participants and their parents for agreeing to participate to this study. We also would like to thank Dr Amanda Ross, who is a post-doctoral biostatistician at the Swiss TPH, for her guidance to perform the survival analysis.
